# Supplementary material for: TP53 Targeted Deep Sequencing of Cell-Free DNA in Esophageal Squamous Cell Carcinoma Using Low-Quality Serum: Concordance with Tumor Mutation
Source: Int J Mol Sci. 2021 May 26;22(11):5627. doi: 10.3390/ijms22115627 (PMC8197963; doi:10.3390/ijms22115627)

Supplementary table S1: List of *TP53* variants detected in esophageal tumor FFPE tissue which were not detected in serum cfDNA

Variant coordinate	Protein variant	Mutation effect	Domain function	REVEL score
7579458G>T	P77T	missense	SH3-like/Pro-rich	0.305
7579456T>G	P77P	silent	SH3-like/Pro-rich	---
7578467T>G	T155P	missense	DNA binding	0.581
7578444G>A	I162I	silent	DNA binding	---
7578413C>T	V173M	missense	DNA binding	0.892
7578413C>A	V173L	missense	DNA binding	0.88
7578397T>G	H178P	missense	DNA binding	0.918
7578263G>A	R196*	nonsense	DNA binding	---
7578259A>C	V197G	missense	DNA binding	0.914
7578204A>C	S215R	missense	DNA binding	0.917
7578203C>T	V216M	missense	DNA binding	0.956
7578191A>T	Y220N	missense	DNA binding	0.91
7578177C>T	E224E	splice	DNA binding	---
7577576G>A	N235N	silent	DNA binding	---
7577551C>A	G244C	missense	DNA binding	0.94
7577535C>A	R249M	missense	DNA binding	0.911
7577141C>T	G266E	missense	DNA binding	0.973
7577124C>A	V272L	missense	DNA binding	0.878
7577123A>C	V272G	missense	DNA binding	0.707
7577082C>T	E286K	missense	DNA binding	0.949
7577079C>A	E287*	nonsense	DNA binding	---
7577022G>A	R306*	nonsense	NA	---

Supplementary table S2: List of *TP53* variants were concordantly detected in archival serum cfDNA and esophageal tumor tissue biopsy.

Status	Variant genomic coordinate	Protein variant	REVEL score	Q-value	No. reference reads	No. alternative reads	RVSB	Genomic position error	Mutation effect	Domain function	Mean coverage
Case	7577045TC>T	p.?	NA	618.6	28385	94	0.58	0.000089	NA	NA	476389.5
Case	7579358C>A	R110L	0.63	34.1	48454	22	0.69	0.000079	missense	DNA binding	425847.5
Case	7578454G>T	A159D	0.84	245.0	27974	62	0.62	0.000087	NA	NA	840911.5
Case	7578469C>A	G154V	0.75	226.3	46902	78	0.51	0.000099	missense	DNA binding	840911.5
Control	7577121G>T	R273S	0.91	51.9	36510	33	0.62	0.000105	missense	DNA binding	523326.5
Control	7577509C>T	E258K	0.96	47.0	40180	65	0.63	0.000409	missense	DNA binding	523326.5
Case	7578525G>C	C135W	0.88	47.6	34450	22	0.57	0.000069	missense	DNA binding	560905.5
Case	7577073G>A	L289F	0.34	82.9	49516	109	0.53	0.000385	missense	DNA binding	641517.5
Case	7578212G>A	R213*	---	114.2	4305	41	0.59	0.000727	nonsense	DNA binding	641517.5
Case	7577535CTCCGGT>C	p.?	NA	1000.0	48848	69	0.77	5.50E-07	NA	NA	813073.5
Case	7577121G>T	R273S	0.91	44.1	21364	20	0.59	0.000105	missense	DNA binding	389311
Case	7578234A>C	Y205*	---	58.1	5571	18	0.58	0.000169	nonsense	DNA binding	389311
Control	7577509C>T	E258K	0.96	52.4	28867	53	0.57	0.000409	missense	DNA binding	592767.5
Case	7577079C>T	p.E287K	0.58	40.0	49625	69	0.70	0.000248	missense	DNA binding	735729
Case	7577536T>A	R249W	0.85	507.2	49234	261	0.52	0.00028	missense	DNA binding	677844
Case	7577556C>A	C242F	0.98	103.9	40386	48	0.54	0.000084	missense	DNA binding	928413.5
Case	7573029G>A	p.?	NA	1000.0	9036	2029	0.64	0.000953	intronic	NA	575176
Case	7577079C>T	p.E287K	0.58	34.3	49690	62	0.58	0.000248	missense	DNA binding	814802
Case	7577018C>T	p.?	NA	202.1	48086	263	0.52	0.000624	splice	NA	732647
Control	7577536T>A	R249W	0.85	37.0	49537	51	0.64	0.00028	missense	DNA binding	908029.5
Control	7577580T>C	Y234C	0.94	69.0	49235	210	0.61	0.001618	missense	DNA binding	908029.5
Control	7577509C>T	E258K	0.96	92.3	42415	98	0.64	0.000409	missense	DNA binding	429553.5

Supplementary table S3. .Frequency of neoantigen formation among *TP53* variants detected in several organ malignancies (Bladder, breast, head&neck, prostate, colorectal, stomach, kidney, liver, and lung) matched with the variants in the Golestan ESCC study.

Available data about neoantigen frequency	Concordant mutation with tumor in cfDNA	Protein variant	Frequency of neoantigen formation	Frequency of reported mutation	Mutation protein affinity	Frequency of HLA subtypes
NA	No	**E224E**	.	.	.	.
NA	No	**E287***	.	.	.	.
NA	No	**H178P**	.	.	.	.
NA	No	**I162I**	.	.	.	.
NA	No	**L289F**	.	.	.	.
NA	No	**N235N**	.	.	.	.
NA	No	**P77P**	.	.	.	.
NA	No	**P77T**	.	.	.	.
NA	No	**R196***	.	.	.	.
NA	No	**R306***	.	.	.	.
NA	No	**S215R**	.	.	.	.
NA	No	**T155P**	.	.	.	.
NA	No	**V272G**	.	.	.	.
NA	No	**Y220N**	.	.	.	.
A	No	**E286K**	0.000081	0.0014197	290.3075	0.0570792
A	No	**G244C**	0.0000459	0.0007744	207.3986	0.0592
A	No	**G266E**	0.0000667	0.0011616	212.9428	0.0574209
A	No	**R249M**	0.0000463	0.0006453	207.443	0.0717923
A	No	**V173L**	0.0000356	0.0011616	208.5529	0.0306836
A	No	**V173M**	0.0000424	0.0012907	178.2108	0.0328753
A	No	**V197G**	0.0000233	0.0003872	255.5718	0.0603116
A	No	**V216M**	0.0000564	0.0010325	163.6288	0.054587
A	No	**V272L**	0.0000252	0.0003872	203.1794	0.0651862
NA	Yes	**A159D**	.	.	.	.
NA	Yes	**C135W**	.	.	.	.
NA	Yes	**E287K**	.	.	.	.
NA	Yes	**L289F**	.	.	.	.
NA	Yes	**R213***	.	.	.	.
NA	Yes	**Y205***	.	.	.	.
A	Yes	**C242F**	0.0000892	0.0014197	159.7191	0.0628105
A	Yes	**E258K**	0.0000211	0.0005163	149.7165	0.0408379
A	Yes	**G154V**	0.0000515	0.0009035	189.5567	0.0569621
A	Yes	**R110L**	0.0000773	0.0012907	179.6394	0.0598606
A	Yes	**R249W**	0.0000261	0.0003872	219.3091	0.0674152
A	Yes	**R273S**	0.0000515	0.0009035	182.714	0.0569566
A	Yes	**Y234C**	0.0000981	0.001936	210.6004	0.0506831

Supplementary Figure S1 : Regression graphs for detected mutations in cfDNA per position and alteration.

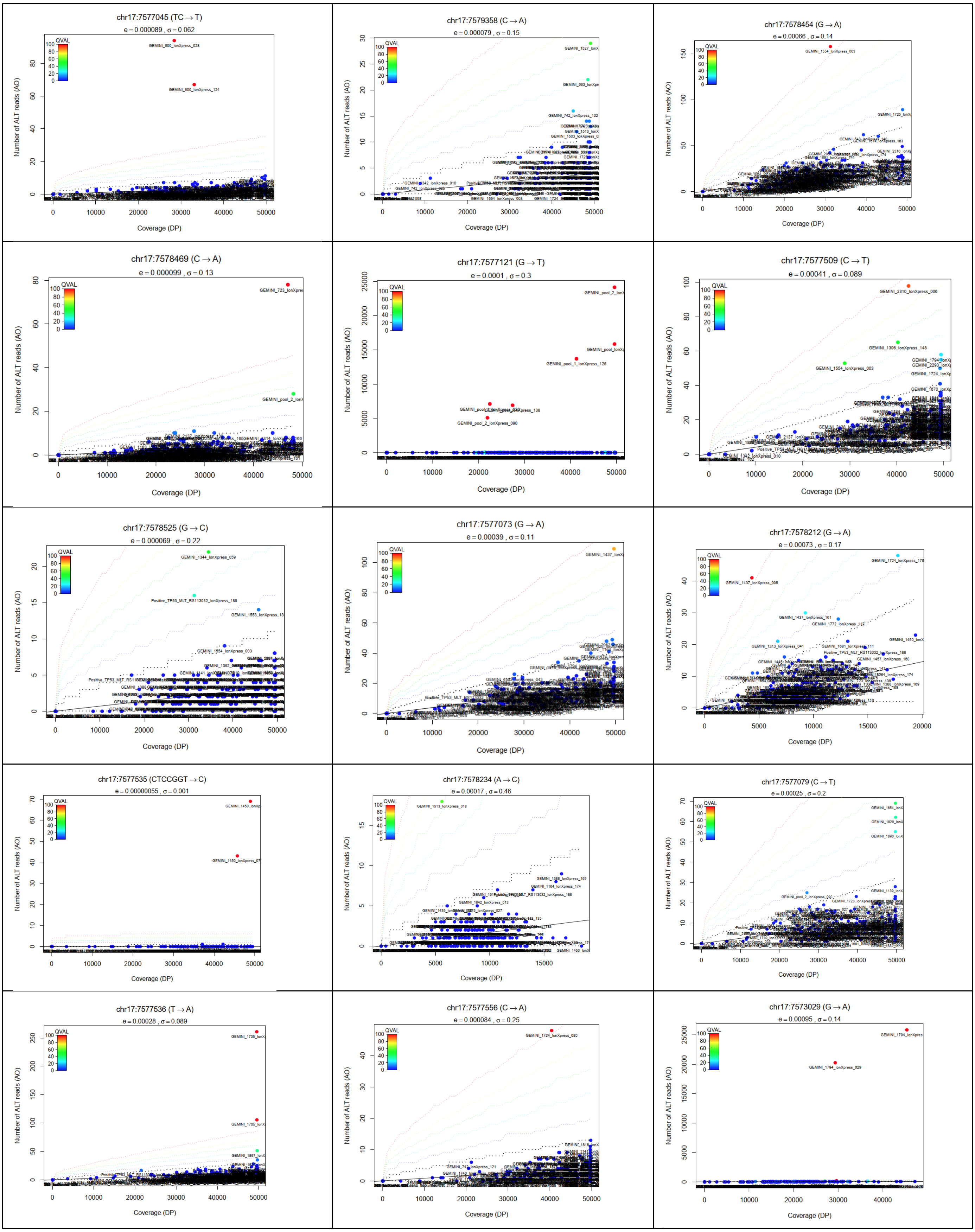

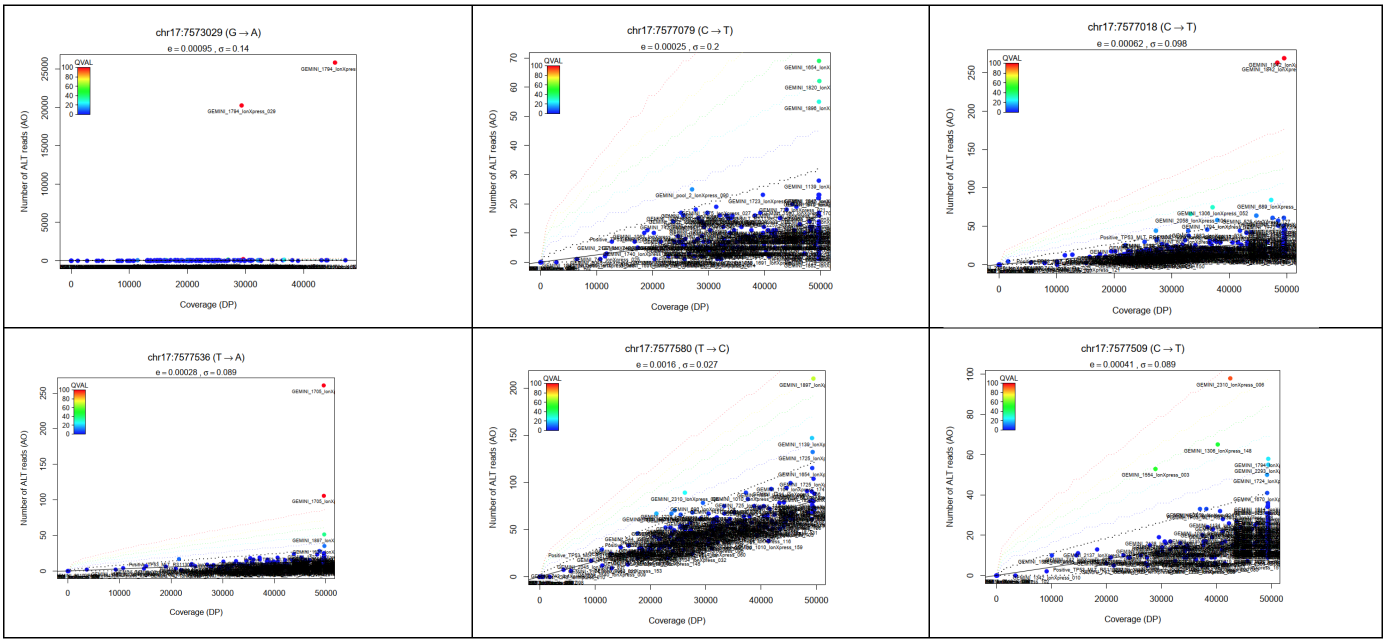

chr17:7573029 (G → A)
e = 0.00095 , σ = 0.14
QVAL
Number of ALT reads (AO)
Coverage (DP)
GEMINI_1794_IonXpress_029
chr17:7577079 (C → T)
e = 0.00025 , σ = 0.2
chr17:7577018 (C → T)
e = 0.00062 , σ = 0.098
chr17:7577536 (T → A)
e = 0.00028 , σ = 0.089
chr17:7577580 (T → C)
e = 0.0016 , σ = 0.027
chr17:7577509 (C → T)
e = 0.00041 , σ = 0.089
GEMINI_2310_IonXpress_006
GEMINI_1306_IonXpress_148
GEMINI_1554_IonXpress_003

Supplementary Figure S2: No statistical difference in HLA distribution of neoantigen formation of tumor *TP53* variants relevant to detectibelity in cfDNA. Boxplot shows higher frequency of neoantigen formations among cfDNA detected *TP53* mutations.

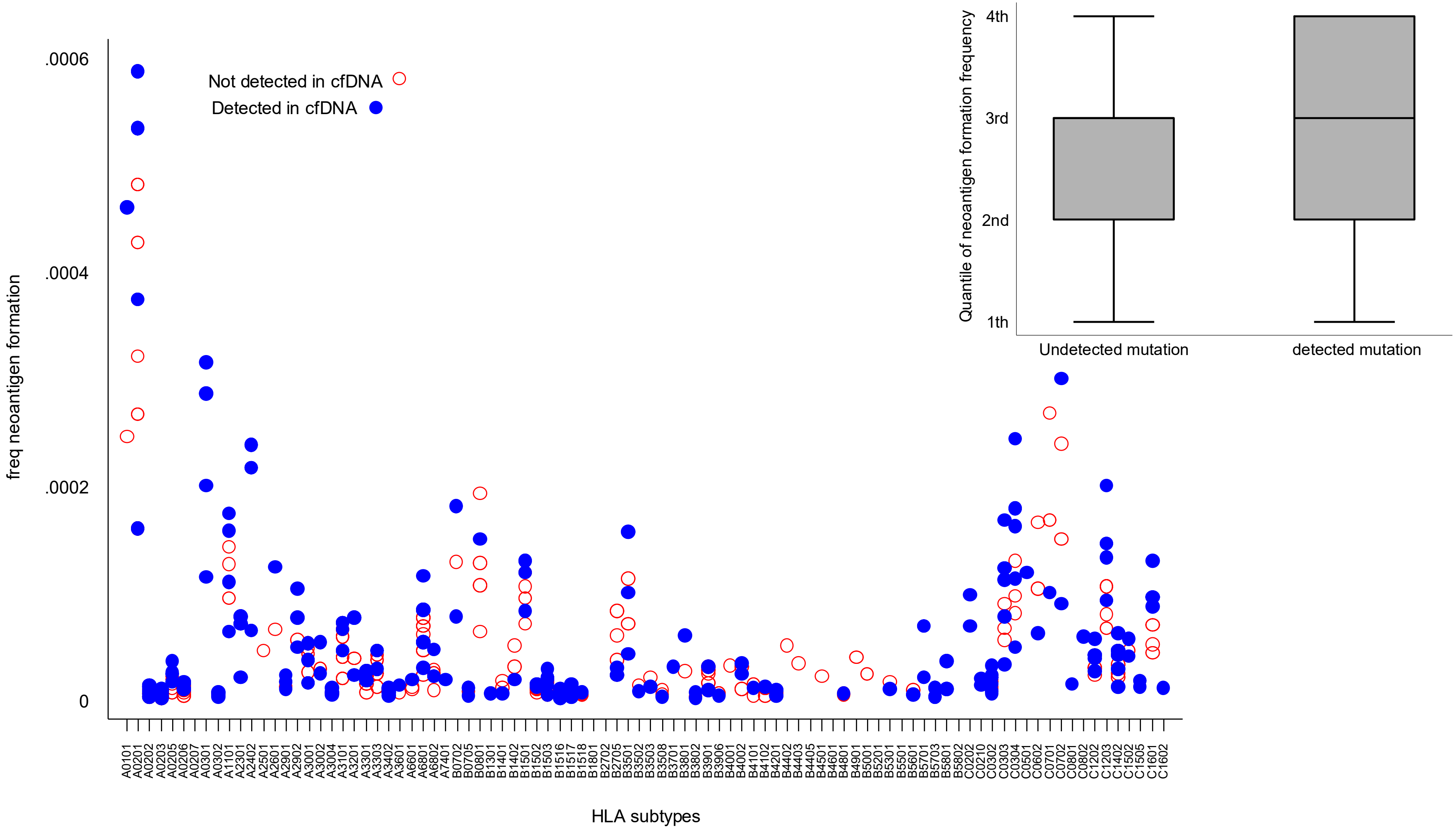

Supplement: Supplementary file 1 [file ijms-22-05627-s001.zip › ijms-1194978-SI.pdf]
